# Identification, In Vitro Testing and Molecular Docking Studies of Microginins’ Mechanism of Angiotensin-Converting Enzyme Inhibition

**DOI:** 10.3390/molecules22121884

**Published:** 2017-12-05

**Authors:** Fernanda C. R. Paiva, Glaucio Monteiro Ferreira, Gustavo H. G. Trossini, Ernani Pinto

**Affiliations:** 1 Department of Clinical and Toxicological Analysis, School of Pharmaceutical Sciences, University of São Paulo, Av. Prof. Lineu Prestes, 580, Bl. 17, CEP 05508-000 São Paulo, Brazil; 2 Department of Microbiology, Institute of Biomedical Sciences, University of São Paulo, Av. Prof. Lineu Prestes, 1374, CEP 05508-000 São Paulo, Brazil; fcrpaiva@usp.br; 3 Department of Pharmacy, School of Pharmaceutical Sciences, University of São Paulo, Av. Prof. Lineu Prestes, 580, Bl. 13, CEP 05508-000 São Paulo, Brazil; gmf@usp.br (G.M.F.); trossini@usp.br (G.H.G.T.)

**Keywords:** cyanobacteria, *Microcystis*, microginin, ACE inhibition, virtual screening

## Abstract

Cyanobacteria are able to produce a wide range of secondary metabolites, including toxins and protease inhibitors, with diverse biological activities. Microginins are small linear peptides biosynthesized by cyanobacteria species that act against proteases. The aim of this study was to isolate and identify microginins produced by the LTPNA08 strain of *Microcystis aeruginosa*, as well as to verify their potential to inhibit angiotensin-converting enzyme (ACE; EC. 3.4.15.1) using in vitro and in silico methods. The fractionation of cyanobacterial extracts was performed by liquid chromatography and the presence of microginins was monitored by both LC-MS and an ACE inhibition assay. Enzyme inhibition was assayed by ACE with hippuryl-histidyl-leucine as the substrate; monitoring of hippuric acid was performed by HPLC-DAD. Isolated microginins were confirmed by mass spectrometry and were used to carry out the enzymatic assay. Molecular docking was used to evaluate microginin 770 (MG 770) and captopril (positive control), in order to predict similar binding interactions and determine the inhibitory action of ACE. The enzyme assay confirmed that MG 770 can efficiently inhibit ACE, with an IC_50_ equivalent to other microginins. MG 770 presented with comparable interactions with ACE, having features in common with commercial inhibitors such as captopril and enalaprilate, which are frequently used in the treatment of hypertension in humans.

## 1. Introduction

Some cyanobacterial species can produce different types of bioactive peptides (cyanopeptides), such as aeruginosins, cyanopeptolins, microginins, microviridins, anabaenopeptins and microcystins. These secondary metabolites normally contain non-proteinogenic amino acids, and are biosynthetized via non-ribosomal polypeptide synthetases (NRPS), or via mixed routes such as polyketide synthase (PKS) and NRPS [1,2]. These secondary metabolites have high structural diversity and thus, different biological activities [3]. Studies over the last few decades have identified substances with cytotoxic, antifungal, antibacterial, antiviral and anti-inflammatory actions [4], as well as potent inhibitors of various proteases [5]. Microcystins are well known in the scientific literature as hepatotoxins that inhibit phosphatases type 1 and 2A. Microginins are reported to be inhibitors of proteases, including angiotensin-converting enzyme (ACE), and thus, are important candidates for the development of anti-hypertensive compounds [6]. However, the mechanisms of action of microginins have not been fully elucidated [7]. Microginins were first reported in the literature in 1993 by Okino et al. [8]; since then, at least 34 variants have been reported [9]. Structurally, microginins are linear peptides composed of three to six amino acid residues with molecular weights ranging from 574 to 930 Da. In the N-terminal portion, these molecules present a derivative of decanoic acid, 3-amino-2-hydroxydecanoic acid (Ahda), and a predominance of two tyrosine units in the C-terminal portion [10]. Nevertheless, the physiological functions of these compounds have not been fully elucidated. Therefore, the investigation of cyanopeptides is relevant because of the ecotoxicological effects as well as their pharmacological potential. The aim of this study was to isolate and identify microginins produced by the LTPNA08 strain of *Microcystin aeruginosa*, and to verify their potential to inhibit ACE (EC. 3.4.15.1), using in vitro and in silico methods. Protocols for isolation and identification of microginins are described herein, as well as an alternative spectrophotometric method for their quantification, based on tyrosine ε. Further, the results indicated that microginins inhibit ACE in a similar mechanism to that described for captopril, suggesting they are potential candidates for the control of arterial hypertension.

## 2. Results

**Figure 1:**
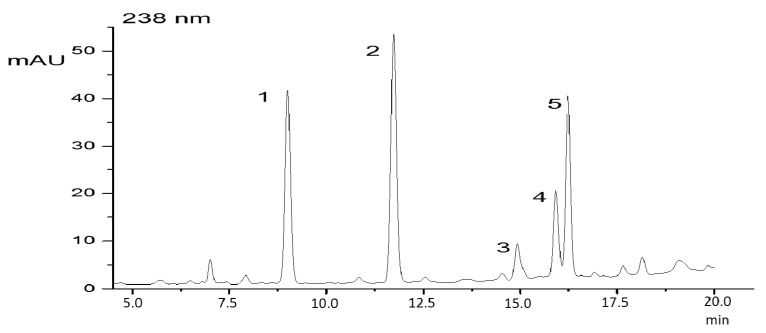
HPLC-DAD elution profile of a methanolic extract of *Microcystis aeruginosa* LTPNA 08. (**1**) MC-RR; (**2**) unknown peak; (**3** and **4**) microginins (MG756 and MG770); (**5**) MC-LR. The chromatographic conditions are described in the Materials and Methods section.

Using the protocol for the extraction of peptides with methanol, an extract was obtained which showed two microginins on reversed phase chromatography (Figure 1), which were identified by their characteristic UV spectra. The UV spectrum with a maxima wavelength at 224 nm and 276 nm is typical for peptides that contain aromatic amino acids. The strain used in this study has been characterized previously by LC-MS [9].

**Figure 2:**
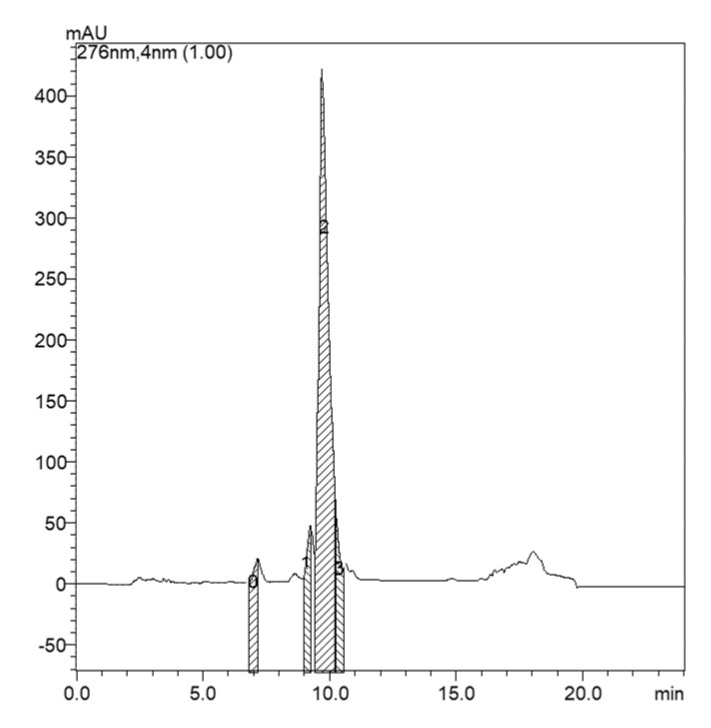
Chromatogram of the SPE fraction of washing with 60% methanol, for the strain LTPNA 08. (**0**) MC-LR; (**1** and **2**) microginins (MG756 and MG770); (**3**) end of the major microginin peak. Peak number 2 shows the fraction that was isolated.

It was verified that the microginin peaks were found in the 60% methanol washes fraction. Thus, from this fraction, the insolation was carried out (Figure 2).

**Figure 3:**
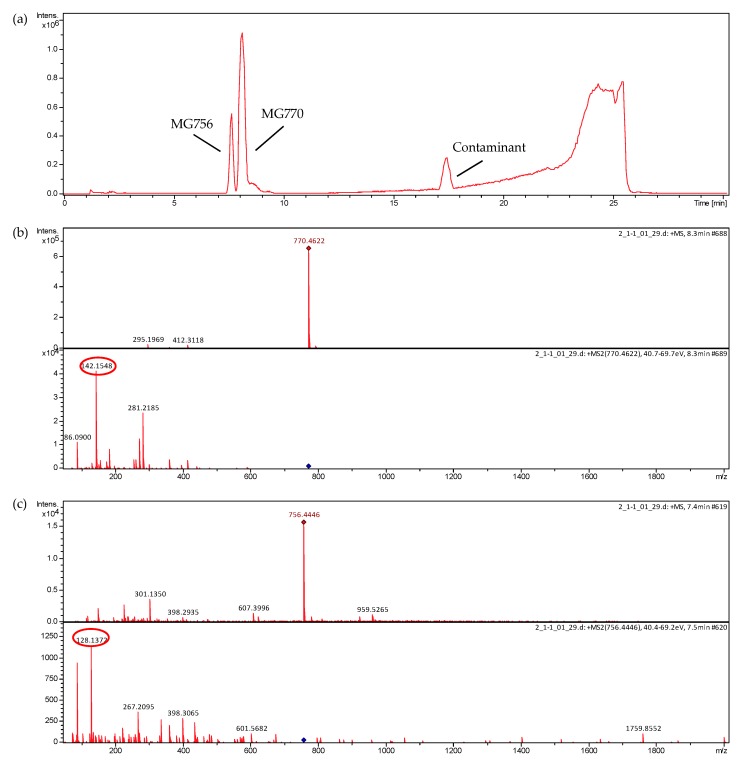
Chromatogram (full scan mode) and MS/MS spectra of microginin fraction post-isolation. The spectra were obtained in a Micro-QTOFII mass spectrometer. The product ions of *m*/*z* 142 and 128, characteristic of microginins, are circulated. The operation conditions are described in the materials and methods section. (**a**) Full scan MS chromatogram; (**b**) MS/MS spectrum of MG 756; and (**c**) MS/MS spectrum of MG 770.

The analysis revealed higher production of microginin *m*/*z* 770 (MG770) than microginin *m*/*z* 756 (MG756) by the strain LTPNA 08. Because they had very close retention times, total microginin separation was partially achieved. These microginins were purified by semipreparative chromatography, and a fraction containing MG 770 (83% pure) was obtained (Figure 3), the peak area of the chromatogram is proportional to the concentration of the substance. The MS/MS spectra of *m/z* 756 and *m*/*z* 770 contain ions at *m*/*z* 128 and *m*/*z* 142, respectively (Figure 3). Enzyme assays for the inhibition of ACE were performed with this enriched fraction of MG 770.

**Figure 4:**
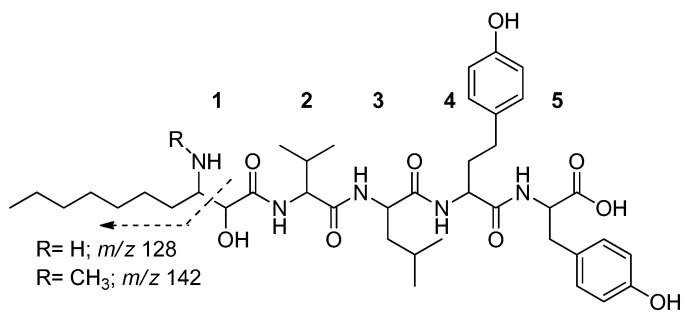
Structure of microginins MG756 (R = H) and MG770 (R = CH3). Adapted from Carneiro et al. [9], where: (**1**) 3-amino-2-hydroxydecanoic acid; (**2**) valine; (**3**) leucine; (**4**) homotyrosine; and (**5**) tyrosine.

The quantification of microginins was not possible due to the lack of an analytical standard (none are commercially available). However, an estimation was performed using spectrophotometry based on similarities between microginins and tyrosine. The choice of tyrosine was based on the presence of a tyrosine and a homotyrosine in the structure of microginin molecules (Figure 4). These amino acids have the same ε, since they have the same chromophore in the aromatic ring; therefore, indirect quantification by spectrophotometry is possible using pure tyrosine. This strategy is useful for fractions less than 1.0 mg, when gravimetrically weighting is not possible.

Microginin inhibits ACE with an IC_50_ of 3.33 µg·mL^−1^. The results showed that the ACE inhibitory rate of microginin was 92.15 ± 2.76% in the concentration of 800 µg·mL^−1^, and the inhibitory rate of captopril was 88.25 ± 1.19% in the concentration of 0.8 µM.

## 3. Discussion

In a typical purification process, cyanobacterial cells are extracted and the resulting extract is concentrated and purified by sample separation techniques, although the number of steps and methods employed varies greatly. In this work, aqueous methanol was used for the extraction of cyanopeptides. After, the analytes were extracted by a C-18 solid-phase extraction (SPE) cartridge for the removal of pigments and large interfering molecules, and also for a previous separation of the compounds in the sample [11]. After verified that the microginin peaks were found in the 60% methanol washes fraction, the tentative of insolation MG 770 was carried out (Figure 2). In total, 4.8 mg of microginins were obtained by semi-preparative HPLC from 3 g of dried strain. This value is consistent with the isolation yield obtained by other authors; Okino et al. [8] isolated 36 mg of microginin from 27 g of dry biomass of cyanobacteria, while Ishida et al. [7] isolated 19.4 mg of 27.6 g and 26.8 mg of 75.3 g. Peptides from cyanobacteria are of pharmaceutical and biological interest due to their enzyme-inhibiting activities [12]. Microginins were initially characterized as inhibitors of zinc metalloproteinases, such as ACE [8] and leucine aminopeptidase (LAP) [13]. The method proposed by Wang et al. [14] for ACE bioactivity assays is convenient, rapid, able to be carried out in isocratic conditions in HPLC-DAD and promotes good separation of the compounds.

Microginins when subjected to collision-induced dissociation in sequential mass spectrometry experiments (CID-MS/MS) present ions of *m*/*z* 128 or *m*/*z* 142 in their fragmentation spectrum (Figure 4) [10]. These ions originate from bond dissociation between C2 and C3 from Ahda, being considered as diagnostics of this class of compounds and, therefore, can be used in the identification of possible structural variants [9].

Relationships between structure and activity showed that C-terminal dityrosine, the amino groups and the hydroxyl Ahda of microginins, play an important role in inhibiting ACE [7]. Furthermore, tetrapeptide microginins tend to be more active than pentapeptide microginins and their variants [7]. However, pentapeptide microginins, such as microginins 478, T1 and T2, present with inhibitory activity at a concentration of about 10 mM [7]. MG770 and MG756 are pentapeptide microginins and the results of the enzyme activity assay performed in the current study are shown in Table 1.

ACE inhibitors are usually small molecules of less than six amino acids, and have hydrophobic amino acids in their structure. In addition, the terminal C and N moieties generally consist of aromatic amino acids and branched aliphatic amino acids, respectively [17]. Microginins present with the predominance of two tyrosine units in the C-terminal portion [10], and MG770 and MG756 possess a tyrosine and a homotyrosine in their C-terminals. K-26, an ACE inhibitor produced by actinomycete, has a partial structure of dityrosine and K-13, another natural ACE inhibitor isolated from *Micromonospora halophytica*, is also a dityrosine derivative [7]. The sequence of amino acids that comprises the structure of a microginin is directly linked to its enzyme inhibition ability. Studies show that the structural variant and its stereochemical conformation are responsible for the inhibition activity [7]. For example, microginins 299-A, -B, -C and -D are inhibitors of leucine aminopeptidase M (LAM); however, they do not inhibit ACE, papain, trypsin, thrombin, plasmin, chymotrypsin or elastase [13,18]. Microginins 99-A and 99-B, and microginins 478 and 51-B, do not inhibit LAM at 100 μg·mL^−1^ [7]. Microginin FR1 showed inhibitory activity for ACE at 16 μg·mL^−1^ [12]. However, the comparison of these inhibitory activities should be considered cautiously, since the activities may be influenced by differences in the assay methods. Our IC_50_ results showed that MG 770 efficiently inhibits ACE, with activity comparable to that of other previously described microginins.

**Figure 5:**
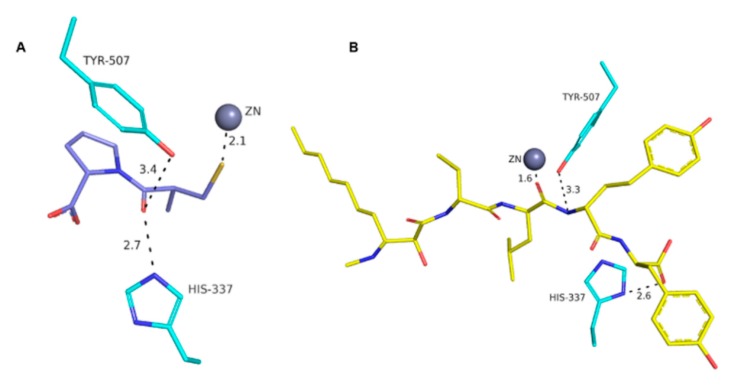
Active site of the ACE and major interactions between: (**a**) captopril co-crystal structure (PDB: 2X8Z); and (**b**) best docked pose of MG 770.

**Figure 6:**
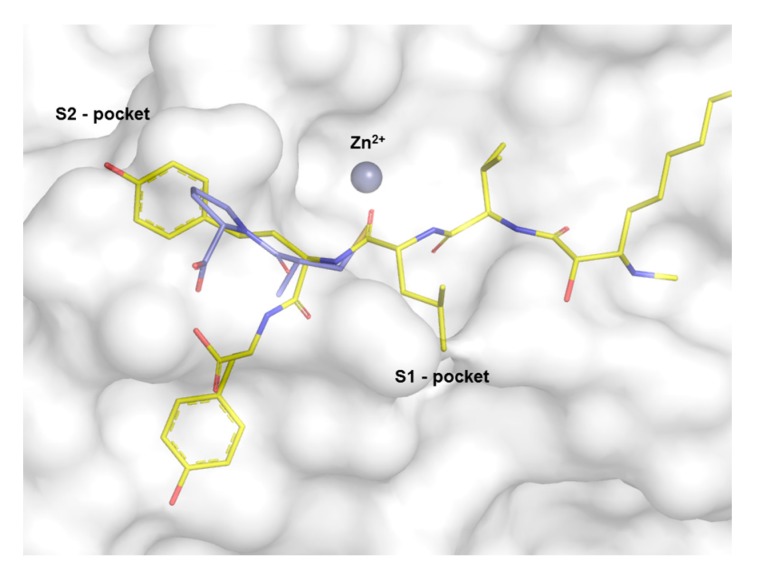
Comparison of captopril crystal structure shown in blue (PDB: 2X8Z) with MG 770 shown in yellow, with regard to cash-outs zinc atom.

Molecular docking studies were performed in order to investigate the possibility of an interaction between MG770 with the active site of ACE. Knowing that MG770 inhibits ACE, the distances of the major interactions in the active site of the enzyme suggest the mechanism of action. The best docking pose of MG770 obtained from the docking studies showed the same interactions than captopril in the active site of the ACE. The crystal structure of the ACE co-crystalized as captopril (PDB: 2X8Z) can be observed from the interaction between the sulphur atom from captopril and zinc, with distances of 2.1 Å (Figure 5A). Docking studies showed an interesting interaction between zinc and the carbonyl homotyrosine, from microginin, at 1.6 Å (Figure 5B). These results suggest an interaction between zinc and molecules (captopril and MG 770), because the carbonyl and thiol are isosters. On the other hand, the residues Tyr507 and His337 show a hydrogen bond with captopril and microginin (Figure 5), and the rings from captopril and tyrosine from microginin were located in the S2-pocket of the ACE (Figure 6); this suggests molecular complementarity in the active site of ACE.

The analysis of the molecular complementarity of MG 770 and ACE suggests two hydrophobic interactions. One in the S1-pocket and another between the terminal residue of the microginin molecule, Ahda. Neumann et al. [12] observed an accessory pocket of the ACE. In addition, other studies have found that FR1 microginin presents with the same interactions as MG 770. Thus, there is a hydrophobic group in the structure of microgynin; this property is due to the presence of a leucine, and it allows the microginin to interact in the S1 pocket.

To better understand the accessory interaction point of the Ahda residue and ACE, we performed interaction field analyses using the GRID program [19]. This analysis can theoretically support and help to understand the Ahda interaction, and provides information about the hydrophobic fields in the active site of ACE, in the same regions observed for MG 770.

## 4. Materials and Methods

### 4.1. Strain Maintenance

The strain used in this study (*Microcystis aeruginosa* LTPNA08) belongs to the culture collection of the Laboratory of Toxins and Natural Products of Algae (LPTNA; University of São Paulo). It was maintained in ASM-1 medium, as described by Gorham et al. [20], at 25 ± 1 °C, with a 12 h light/dark cycle, photon flux of 22 µmol photons m^−2^ s^−1^, initial pH 8.0, and without aeration. At the end of each experiment, i.e., stationary phase of growth, the material was lyophilized and used for biochemical analyses.

### 4.2. Cyanopeptide Identification

Cellular material was obtained from cultures after centrifugation and lyophilization of the pellet. Dried cells from each strain were extracted with aqueous 75% methanol. The material was centrifuged at 4000× *g* for 6 min at 4 °C. The supernatant was collected. Analyses were performed on a Shimadzu Prominence semi-preparative HPLC instrument (Shimadzu, Kyoto, Japan). Separations were achieved using a Luna C18 column (250 mm × 3 mm, 5 μm; Phenomenex^®^, Torrance, CA, USA) protected with a guard column of the same material. Samples were eluted using mobile phase A (water, 0.05% trifluoroacetic acid) and mobile phase B (acetonitrile, 0.05% trifluoroacetic acid). The analysis was conducted on a gradient according to the methodology described by Lawton et al. [21]. Chromatograms were monitored at 276 nm λ_max_ and from 200 to 700 nm in the DAD detector.

### 4.3. Isolation and Purification of Microginins

Twenty liters of the LTPNA 08 strain were grown. After one month of cultivation, the material was centrifuged (4000× *g* for 6 min) and the cell pellet was lyophilized, yielding 3 g of dry biomass. Since the strain of *Microcystis* LTPNA 08 produces both microginins and microcystins, the classical method reported by Lawton et al. [21] was adapted to reliably separate these peptides. A pre-purification was performed on a solid phase extraction column from the crude extract. Dried material was extracted three times with 75% methanol. The extract was eluted with a gradient of 20%, 40%, 60%, 80% and 100% methanol to obtain fractions of different polarities, and these fractions were analysed in HPLC-DAD, according to the methodology described by Lawton et al. [21]. In order to isolate the microginins on a semi-preparative scale (Luna C18(2); 250 mm × 10 mm, 5 μm, Phenomenex^®^), the method described by Lawton et al. was optimized. The best optimized gradient reduced the running time from 55 min to 24 min, using 100% methanol as mobile phase B. 0.1% formic acid was acidifying the water and the methanol. These conditions were used for isolation of microginins in the semiprep column. Analyses were carried out in a Shimadzu Prominence system equipped with a LC-20AT quaternary pump and a SPDM20A photodiode array detector.

### 4.4. Indirect Spectrometric Quantification of Microginins with Tyrosine

The concentrations of the two fractions of microginins were determined spectrophotometrically based on the quantitation of the l-tyrosine amino acid (99% purity; Sigma-Aldrich, Saint Louis, MO 63103, USA). Solutions were measured in a spectrophotometer (Evolution 260 Bio, Thermo Scientific^®^, Bremen, Germany). UV-absorption was used to estimate the concentration of microginins using the Beer-Lambert Law: *A* = *ε*. *l*. *c*: where *A* is absorbance; *ε* is the molar absorptivity/molar absorption coefficient for tyrosine 1 M in NaOH 0.1 M, value defined as 2291.4; *l* is the length of solution light passes through, in this case, cuvette of 1 cm; and ***c*** is the concentration in M [22]. Calibration curves were constructed by analysing the absorbance of tyrosine solutions at concentrations of 1 mM, 0.4 mM, 0.1 mM and 0.01 mM in 0.1 M NaOH. The wavelength used was 293 nm, λ_max_ of tyrosine; 0.1 M NaOH solution was used as blanks. The fractions of dried microginins were resuspended in 5 mL MeOH 100%. 200 μL of the fractions were dried under N_2(g)_ and resuspended in 1.5 mL of 0.1 M NaOH. Samples were measured in the same λ_max_ of Tyr. Samples and Tyr solutions were analysed in triplicate, and the average of the absorbance values was used to calculate the concentration. The calculation used for the indirect quantification was based on microginin absorbance against the calibration curve for Tyr. A factor of two was applied to correct microginin concentration since it possess two chromophores (Tyr itself and homoTyr). The result was expressed in mg of microginins of tyrosine equivalents.

### 4.5. LC-MS Analyses

The fractions obtained were analysed by liquid chromatography mass spectrometry (LC-MS). Analyses were carried out on a Shimadzu Prominence Liquid Chromatography system coupled to a quadrupole time-of-flight mass spectrometer (Micro-TOFQII; Bruker Daltonics, Billerica, MA, USA) with an ESI as the ion source. Separations were achieved using a Fusion-RP column (150 mm × 2 mm, 4 μm; Phenomenex^®^) protected with a guard column of the same material. Samples were eluted using a mobile phase A (water, 0.1% formic acid and 5 mM ammonia formate) and a mobile phase B (acetonitrile), at a flow rate of 0.2 mL/min. The ionization source conditions were as follows: positive ionization; capillary potential of 4000 V; temperature and flow of drying gas (nitrogen) of 5 L/min and 200 °C, respectively; nebulizer pressure of 35 psi. Mass spectra were acquired using full scan mode over the range of *m*/*z* from 50 to 1600.

### 4.6. Enzyme Assay

The ACE inhibiting capability was evaluated according to the method described by Wang et al. ([14], which employs the hippuryl-histidilleucina tripeptide (Hip-His-Leu or HHL) as the substrate and evaluates the formation of hippuric acid (HA) by liquid chromatography (HPLC-DAD). Briefly, the commercial enzyme ACE and the substrate HHL (Sigma-Aldrich^®^) were dissolved in borate buffer (NaOH and boric acid) 100 mmol·L^−1^, pH 8.3, supplemented with 300 mmol·L^−1^ NaCl; their concentrations were 0.2 U/mL and 5 mmol·L^−1^, respectively. Meanwhile, HA and captopril (standard ACE inhibitor) were dissolved in distilled water. The enzyme was pre-incubated in the presence or absence of inhibitor (isolated microginins or captopril) for 30 min at 37 °C, and the reaction was initiated by addition of substrate. After 30 min, the reaction was stopped by adding 250 μL of hydrochloric acid, and the sample was directly injected into the chromatographic system. The concentrations of the inhibitors chosen for the experiment were: 0.1 μM, 0.2 μM, 0.4 μM and 0.8 μM for captopril (Sigma^®^; IC_50_ = 0.021 ± 0.013 μM) and 5 μg·mL^−1^, 10 μg·mL^−1^, 50 μg·mL^−1^, 100 μg·mL^−1^, 500 μg·mL^−1^ and 800 μg·mL^−1^ for microginins. The separation was performed in a reversed phase column (Luna C18(2); 150 nm × 4.6 mm, 5 μm; Phenomenex^®^) using (A) water with 0.05% trifluoroacetic acid (TFA) and 0.05% triethylamine, and (B) acetonitrile as mobile phase; the ratio of solvent A:B was 7:3. The isocratic elution was used and the formation of HA was monitored at 226 nm. The reaction system was: 10 μL HHL (5 mmol·L^−1^), 10 μL ACE (0.2 U/mL), 40 μL inhibitor and 40 μL borate buffer 100 mM (pH = 8.3). The buffer was incubated in the thermomixer for 5 min at 37 °C, the inhibitor (isolated microginins or captopril) and the enzyme were added. After 5 min, the substrate was added to start the reaction.

The inhibitory rate was calculated, according to Wang et al. [14], by I% = (A − B)/A × 100%; where A is the peak area of HA without adding ACE inhibitors; and B is the peak area of HA with the addition of ACE inhibitors [14]. All data presented are the average of three repeats; the data are presented as means ± SEM.

### 4.7. Molecular Docking

The 3D-structure of the peptide MG 770 considered for this study was the linear pentapeptide MeAhda-l-Val-l-Leu-l-HTyr-l-Tyr (where MeAhda is the *N*-terminal [2*S*,*3R*]-3-methyl-amino-2-hydroxy-decanoic-acid and HTyr is homotyrosine) and the configuration of amino acids was based on previous reports described in the literature [10,13]. MG 770 3D-structure was optimized by energy minimization using steepest descent and conjugate gradient techniques. The crystal structures of the human ACE-captopril complex (2X8Z.pdb) were derived from the RCSB Protein Data Bank (PDB) (http://www.rcsb.org/pdb/home/home.do). Before the docking, ligand and water molecules were removed from the binding pocket and hydrogen atoms were added in standard geometry using the Biopolymer module implemented in the platform SYBYL 2.1, whereas zinc atoms (cofactor) were retained in the ACE model. Finally, the polar hydrogens were added to the ACE model. Docking was performed using the program GOLDv5.0. The docking runs were carried out with a radius of 8 Å, with coordinates by zinc atom enzyme ACE. The best ranked docking pose of the peptide in the active site of ACE was determined according to the scores and binding-energy values.

The molecular interaction fields were viewed and treated in PyMOL 1.3 software (version 1.3, Schrödinger, Cambridge, MA, USA), where images of each chemical probe interacting with their respective crystallographic structure were generated. The structure (PDB code: 2X8Z) was used as a display type for fields generated from all the crystallographic structures. The data were compared and analysed in order to pursue certain standards of chemical properties in each subsite of the ACE-captopril complex.

## 5. Conclusions

The cyanobacterial strain *Microcystis aeruginosa* LTPNA 08 can produce microginins 770 and 756. The protocol described herein allowed the isolation of a fraction containing MG 770 (83% pure). A spectrophotometric assay based on the indirect quantification of MG 770 with pure tyrosine is efficient to estimate the weight of fractions with less than 1 mg of microginins, and containing Tyr or HTyr. The enzyme assay confirmed that MG 770 can efficiently inhibit ACE, with an IC_50_ equivalent to other microginins. Using virtual screening, MG 770 presented with comparable interactions with ACE, having features in common with commercial inhibitors such as captopril and enalaprilate, which are frequently used in the treatment of hypertension in humans. Microginins can be considered candidates for the development of new pharmaceuticals, since they can be easily obtained from either cyanobacterial cultivation and isolation or organic synthesis.

## Figures and Tables

**Table 1:** Microginins’ inhibitory activities of ACE.

Peptide	IC_50_ (µg·mL^−1^)	Reference
MG 478	10	[7]
MG 51-A	>100	[7]
MG 51-B	>100	[7]
MG 91-A	>100	[7]
MG 91-B	>100	[7]
MG	7	[8]
MG T1	5	[15]
MG T2	7	[15]
A-Mg 478	700	[7]
MG 770	3.33	Current study
Cyanostatins A *	110	[16]
Cyanostatins B *	130	[16]

* Structurally related to the microginins.
